# Impact of a Tutored Theoretical-Practical Training to Develop Undergraduate Students’ Skills for the Detection of Caries Lesions: Study Protocol for a Multicenter Controlled Randomized Study

**DOI:** 10.2196/resprot.7414

**Published:** 2017-08-16

**Authors:** Mariana Minatel Braga, Tathiane Larissa Lenzi, Fernanda Rosche Ferreira, Fausto Medeiros Mendes, Daniela Prócida Raggio, José Carlos Imparato, Marcelo Bonecker, Ana Carolina Magalhães, Linda Wang, Daniela Rios, Juliano Pelim Pessan, Cristiane Duque, Maria Augusta Bessa Rebelo, Ary Oliveira Alves Filho, Marina De Deus Moura Lima, Marcoeli Silva Moura, Alessandro Diogo De Carli, Mariane Emi Sanabe, Maximiliano Sergio Cenci, Elenara Ferreira Oliveira, Marcos Britto Correa, Rachel Oliveira Rocha, Julio Eduardo Zenkner, Pedroza Uribe Murisí, Stefania Martignon, Juan Sebastian Lara, Fatima Gabriela Aquino, Alfredo Carrillo, Chun Hung Chu, Chris Deery, David Ricketts, Paulo Melo, José Leopoldo Ferreira Antunes, Kim Rud Ekstrand, IuSTC Group

**Affiliations:** ^1^ Dental School Pediatric Dentistry Departament University of São Paulo Sao Paulo Brazil; ^2^ Initiatives for undergraduate Students’ Training in Cariology (IuSTC Group) Multi-institutional group São Paulo Brazil; ^3^ Dental School Federal University of Santa Maria Santa Maria Brazil; ^4^ Dental School Federal University of Minas Gerais Belo Horizonte Brazil; ^5^ CPO Sao Leopoldo Mandic Campinas, SP Brazil; ^6^ School of Dentistry University of São Paulo Bauru Brazil; ^7^ School of Dentistry São Paulo State University (Unesp) Araçatuba Brazil; ^8^ School of Dentistry Federal University of Amazonas Manaus Brazil; ^9^ Postgraduate Programme in Dentistry Federal University of Piaui Teresina, PI Brazil; ^10^ School of Dentistry Federal University of Mato Grosso do Sul Campo Grande, MS Brazil; ^11^ School of Dentistry “Prof Albino Coimbra Filho” Federal University of Mato Grosso do Sul Campo Grande, MS Brazil; ^12^ Graduate Program in Dentistry Federal University of Pelotas Pelotas, RS Brazil; ^13^ Facultad de Odontologia Universidad de Guadalajara Guadalajara Mexico; ^14^ UNICA - Caries Research Unit Research Vice-rectory, Universidad El Bosque Bogotá Colombia; ^15^ Dental Innovation and Translation Centre King's College of London London United Kingdom; ^16^ UNICA - Caries Research Unit Research Vice-rectory Universidad El Bosque Bogotá Colombia; ^17^ The Dental Health Unit The University of Manchester Manchester United Kingdom; ^18^ Carrera de Odontología Universidad Católica Asunción Paraguay; ^19^ Facultad Autonoma de Asunción Asunción Paraguay; ^20^ Faculty of Dentistry The University of Hong Kong Hong Kong China (Hong Kong); ^21^ University of Sheffield Sheffield United Kingdom; ^22^ Dundee Dental Hospital and School University of Dundee Dundee United Kingdom; ^23^ Faculty of Dental Medicine EpiUnit University of Porto Porto Portugal; ^24^ School of Public Health University of São Paulo São Paulo Brazil; ^25^ Department of Odontology University of Copenhagen Copenhagen Denmark

**Keywords:** active learning, cariology, dental education, laboratory training

## Abstract

**Background:**

Tutored laboratorial activities could be a manner of improving the competency development of students. However, its impact over conventional theoretical classes has not yet been tested. Additionally, different university contexts could influence this issue and should be explored.

**Objective:**

To assess the impact of a tutored theoretical-practical training for teaching undergraduate students to detect caries lesions as compared with theoretical teaching activities. The impact of these teaching/learning activities will be assessed in terms of efficacy, cost/benefit, retention of knowledge/acquired competences, and student acceptability.

**Methods:**

Sixteen centers (7 centers from Brazil and 9 centers from other countries throughout the world) are involved in the inclusion of subjects for this protocol. A randomized controlled study with parallel groups will be conducted. One group (control) will be exposed to a 60- to 90-minute conventional theoretical class and the other group (test) will be exposed to the same theoretical class and also a 90-minute laboratory class, including exercises and discussions based on the evaluation of a pool of images and extracted teeth. The mentioned outcomes will be evaluated immediately after the teaching activities and also in medium- and long-term analyses. To compare the long-term outcomes, students who enrolled in the university before the participating students will be interviewed for data collection and these data will be used as a control and compared with the trained group. This stage will be a nonrandomized phase of this study, nested in the main study. Appropriate statistical analysis will be performed according to the aims of this study. Variables related to the centers will also be analyzed and used to model adjustment as possible sources of variability among results.

**Results:**

This ongoing study is funded by a Brazilian national funding agency (CNPq- 400736/2014-4). We expect that the tutored theoretical-practical training will improve the undergraduate students’ performance in the detection of caries lesions and subsequent treatment decisions, mainly in terms of long-term retention of knowledge. Our hypothesis is that tutored theoretical-practical training is a more cost-effective option for teaching undergraduate students to detect caries lesions.

**Conclusions:**

If our hypothesis is confirmed, the use of laboratory training in conjunction with theoretical classes could be used as an educational strategy in Cariology to improve the development of undergraduate students’ skills in the detection of caries lesions and clinical decision-making.

## Introduction

### Background

There is an imminent demand to prepare dental professionals to not only possess knowledge, but also have developed skills to detect dental caries and make decisions about caries management based upon the current best scientific evidence [1]. Thus, an improved education in Cariology for dental undergraduate students as future practitioners is fundamental for adequate caries diagnoses [2].

A recent systematic review has pointed out that caries diagnosis is the topic within Cariology that has received the most concern in terms of developing training methodologies and/or education for undergraduate students [3]. This suggests not only the importance of the subject within the dental professional training, but also the difficulty involved in successfully preparing professionals to be able to perform this step in the dental clinic.

Although it may appear to be a simple step, the detection of caries lesions, as a part of the diagnosis process, involves several aspects that require not only knowledge about the subject, but also theoretical, clinical, and interpersonal skills. In this sense, practical training may be essential for the development of these specific skills [4]. The laboratory activities systematically provide opportunities for undergraduate students to experience different simulated situations covering a variety of circumstances that they should be able to solve in clinical practice. Moreover, practical training allows more interactivity between students and teachers and/or tutors in the teaching-learning process [5,6], besides providing rapid feedback on their performance [7]; thus, improving the educational outcome.

Several systems for caries detection have been used, to provide important information about the disease and also to guide professionals in making treatment decisions [8]. The International Caries Detection and Assessment System (ICDAS) is one of the options proposed to standardize this process internationally [9]. Although student performance in the detection of caries lesions using this system has already been assessed [10-12], these investigations were performed in small groups of students or in only 1 institutional center, reducing the external validity of these findings [13].

A preliminary study conducted at the University of São Paulo found that the tutored theoretical-practical training seems to improve the caries detection by undergraduate students using ICDAS [14]. However, it was not possible to assess the real impact of this active-learning methodology in the teaching of this field, which involves more time and costs, as compared with the theoretical activity alone. Furthermore, this effect may be dependent on different university contexts, mainly because caries diagnosis content has not been taught in the same way in different curricula. To the best of our knowledge, this is a pioneering multicenter study that aims to investigate the short- and long-term impact of the tutored theoretical-practical training for detecting caries lesions in undergraduate courses in comparison with theoretical activities.

### Objective

The aim of the present protocol will be to evaluate the effect of a tutored theoretical-practical training for teaching undergraduate students to detect caries lesions in comparison with theoretical teaching activities. The impact of these teaching/learning activities will be assessed in terms of efficacy, cost/benefit, retention of knowledge/acquired competences, and student acceptance.

## Methods

### Ethical Aspects

The Research Ethics Committee of all institutions involved previously approved this protocol. The participants will receive and sign an informed consent prior to their involvement in the research. For ethical reasons, the students allocated to the theoretical group will receive laboratory training at the end the outcome assessments. Participant confidentiality will be ensured using identification code numbers. Participant identifiable information will be stored in locked filing cabinets in a secure room.

### Study Design

A multicenter randomized controlled study with parallel groups will be conducted. One group (control) will be exposed to a 60- to 90-minute conventional theoretical class and the other group (test) will be exposed to the same theoretical class along with a 90-minute laboratory class, including exercises and discussions based on the evaluation of a pool of images and extracted teeth.

**Figure 1:**
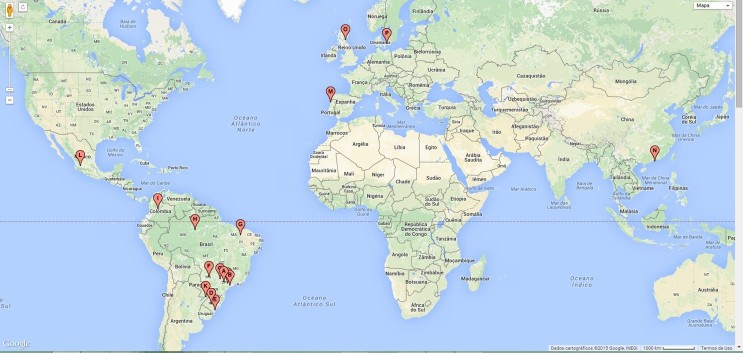
Mapping of the institutions involved in the study.

This study will involve 7 centers from Brazil and 9 centers from other countries throughout the world (Figure 1). The centers represent two distinct contexts: 1 coordinating center, a precursor in the development of the theoretical and laboratory activity that will be tested, while the other institutions represent those that will receive the proposal to develop the same activity with support provided by the precursor center. The inclusion of different centers aims to cover different regions and institutions with different profiles to increase the external validity of the study. As the coordinating center is in São Paulo, Brazil, 6 other institutions were invited to cover different parts of the country. Similarly, the other centers throughout the world are located in different regions, including Latin America (Colombia and Paraguay), Asia (Hong Kong), and Europe (Copenhagen, United Kingdom, and Portugal). Other centers may be optionally incorporated throughout the study, according to interest and judgment of necessity. The Initiatives for Undergraduate Students’ Training in Cariology is a collaborative group made up of all people involved in this study. The detailed roles of each member and respective affiliations are described in Multimedia Appendix 1.

### Training for Operationalization of the Study in Different Centers

The researchers responsible for the study in each center will participate in an initial joint meeting to clarify the methodology to be followed in the study. Training and collaboration of the participants from each center will be made in order to properly adapt the logistics needed for the study, considering the characteristics of each center. The research team will detect all particularities of each center, and a proposal for the implementation of the activity will be jointly developed by the coordinating center and each participating institution. In addition, the principal investigator will train the staff members that will apply the activity at each center.

### Preparation of the Material for Evaluations

The coordinating center will be responsible for organizing and/or assisting in the organization of the material to be used in the theoretical-practical training, including preparation of a pool of images and extracted teeth contemplating different stages of caries severity. The pool of images will be prepared and used in all centers. The teeth will be obtained from the Bank of Human Teeth located at each institution or similar donators of material for this purpose. A tutorial was developed to guide investigators from each center to prepare their samples aided by an investigator from the coordinating center.

### Experimental Groups

Undergraduate students at the beginning (up to second or third year) and the end of the Dentistry course (last 2 years) will be included. All students will be invited to participate, but only those who consent to participate will be included. The classes will be randomly divided into 2 groups. The randomization will be made by a central allocation located in the coordinating center, by sending the list of students to be invited. All students will be randomized, even those that will not participate. These losses will be computed and analyzed later.

One group (control) will be exposed to a 60- to 90-minute conventional theoretical class and the other group (test) will be exposed to the same theoretical class and a 90-minute laboratory class, including exercises and discussions based on the evaluation of a pool of images and extracted teeth. All students will attend the class together before the allocation. For the test group, the participants will be assigned in small groups (7-10 students) mediated by graduate tutors and/or teachers. Students will assess 30 clinical images and 10 teeth for severity (ICDAS scores) and activity status [14]. Tutors/teachers will work on correcting exercises and also discussion of questions raised by the group. In case of disagreement, tooth revaluation will be possible with the help of tutors/teachers so that students understand potential mistakes. For the control group, they will begin the outcomes assessment after having finished the theoretical class.

Despite the allocation, both groups will begin their activities (for training or outcomes assessment) at the same time, avoiding demotivation of any group. Students and teachers/tutors will know the allocated group only when they will be prepared for the activities after the theoretical class, guaranteeing the allocation concealment.

### Outcomes Assessment

#### Cost Efficacy

To evaluate the efficacy of the teaching and learning methods both the theoretical knowledge, and the practical skills and clinical decision-making capacity of the students will be evaluated after the theoretical class (control group) and after the theoretical laboratory training (test group).

To evaluate the theoretical knowledge, the students will answer questions about caries lesions detection prepared by professors with expertise in this field. Furthermore, the participants must understand the impact of the correct decision or error when using the ICDAS in clinical practice. The practical assessment will be made through the evaluation of extracted teeth for 1 minute each. Students will evaluate selected tooth surfaces according to the ICDAS criteria with the aid of a light reflector, a plane buccal mirror, and a ballpoint probe. To simulate the distance to observe teeth in the mouth, teeth will be positioned on a plane surface approximately 30 cm from the examiners’ eyes. The teeth will first be examined wet, and then they will be dried for 5 seconds with compressed air. For this evaluation, the students' responses regarding caries severity will be compared with a template made by teachers after visual inspection. To evaluate the capacity of decision making, the undergraduate students should propose the treatment option based on caries lesion detection for 5 clinical cases. Cases were prepared to simulate usual clinical conditions. To assess the cost efficacy of the methods, the efficacy will be considered the number of true answers registered by the students independently on the theoretical and practical tests, as well as in the decision-making query. Direct and indirect costs will be calculated, both individually (the activity itself) and institutionally (to maintain the necessary structure and provide time for the activity). Cost estimation should include planning, preparation of the material, staff training, and implementation of the activity. Lecture and practical activity costing data will be directly collected at all study sites considering institutional information itself. In addition, national databases will be searched to estimate costs of maintenance of universities in each location. Data from these 2 sources will be collated to produce tables of costs for the activity, center, and country.

#### Perception and Acceptance

The students’ perception of the activity and their degree of satisfaction with the knowledge achieved will be assessed. Questions will evaluate the preparation level, nervousness, satisfaction, and acceptance of students regarding the proposed activities. These outcomes will be evaluated based on the State Trait Personality Inventory Scale [15]. Moreover, the goal of this outcome will verify if the students who participated in the laboratory training felt more prepared for the caries lesions detection in a clinical situation.

#### Retention of Knowledge and Skills

**Figure 2:**
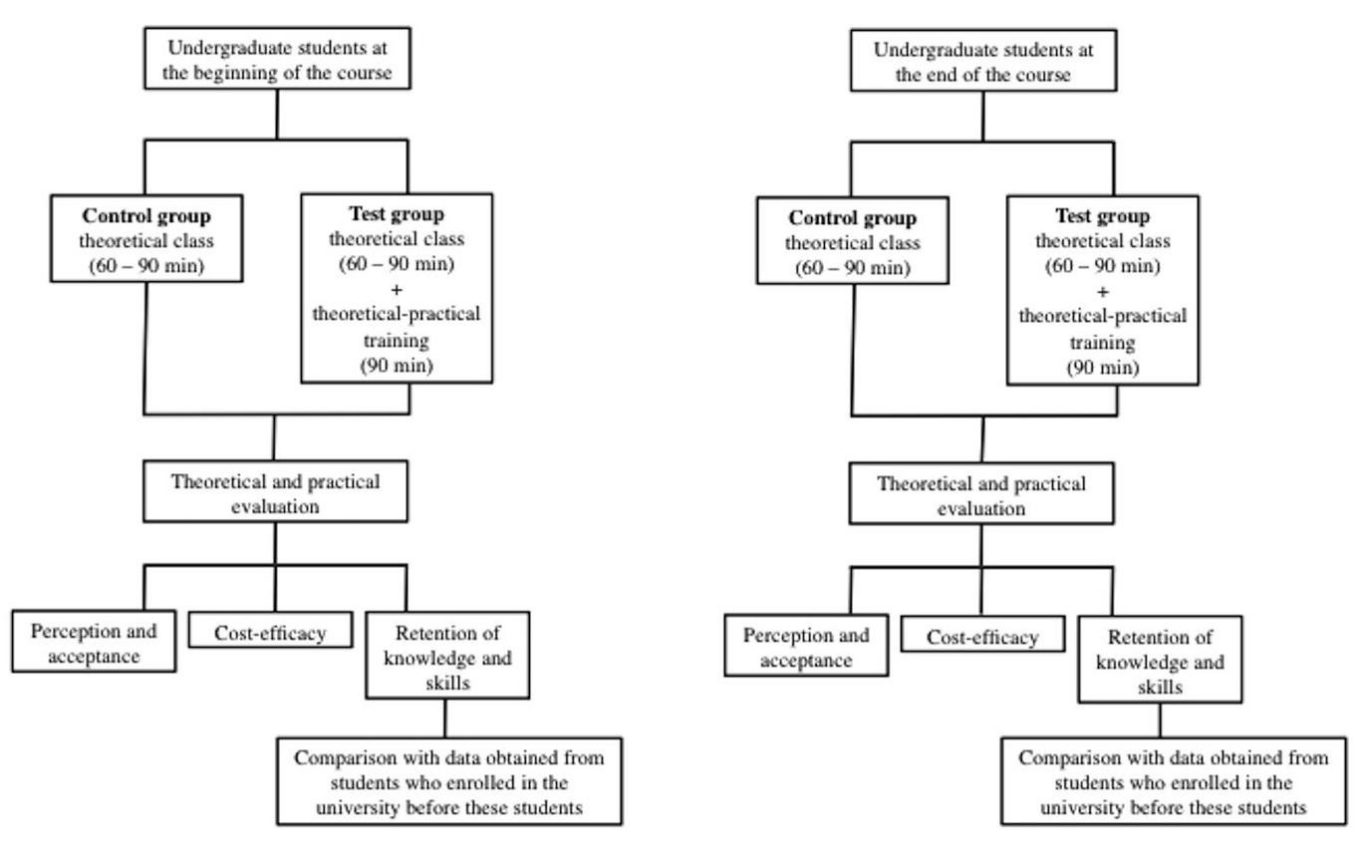
Study design and phases to be conducted.

To evaluate the retention of knowledge and skills acquired after the tutored theoretical laboratory training, students who entered the university before the participating students will be interviewed for data collection, and these data will be used as control and compared with the trained group. This stage will be a nonrandomized phase of the study, nested in the main study. To compare the long-term outcomes, a theoretical and practical assessment will be applied to participating students after 1 or 2 years following the first training and compared with a class in the same year of the undergraduate program, but who had not been exposed to this methodology. This stage of study will be adjusted considering the curriculum of each center in accordance with the course subjects offered in the periods of interest. The flowchart of the study design is summarized in Figure 2.

### Center/Student Characteristics

Some characteristics related to each center will be collected during the planning and execution/data collection stages. In the planning phase, a structured questionnaire will be applied to the center coordinators in order to identify relevant aspects of center size/potential, curricula, human resources/faculty preparation, infrastructure/facilities, and expected difficulties for participant centers. Afterward, in the execution phase, we will first map each student’s background and willingness to learn Cariology and caries detection by a form they should fill out before the theoretical class. The student’s background will show both the student’s knowledge at baseline, and also the possible contents taught at his/her center. Second, qualitative research on topics and strategies used in teaching/learning of Cariology will be investigated among students and lecturers in different centers. These quantitative-qualitative variables will be used as independent variables related to the center in further analysis.

### Statistical Analysis

For the theoretical evaluation, total number of correct responses for test and control groups will be compared at both times of evaluation (immediate and long term). Data from the number of correct responses per question will be submitted to analysis, also considering the question as a factor.

For the practical assessment, performance-related parameters (sensitivity, specificity, accuracy) when using the visual method for the caries detection and evaluation of lesions activity will be calculated. These values will be compared between groups using multilevel analysis, considering both experimental groups and different centers.

The scores given by students for acceptance and perception of the activities carried out will be compared between groups. Regression analysis will be used in each experimental group to verify the association between student performance in caries lesions detection versus theoretical knowledge and student perception in relation to the activity. Baseline student knowledge and willingness to learn caries detection will be considered during these analyses.

The association between the results of immediate theoretical and practical assessments with knowledge and skills retained in the long term will be assessed.

For all analyses, a multilevel approach will be used to consider different levels explored. The assessment, the student, the center, and the country could be considered as possible levels for these analyses considering the possible clustering observed for centers and, eventually, countries. For all the parameters tested, the influence of the different centers and the level in the course (first or end years) will be investigated by regression analysis. The significance level will be set at 5%.

## Results

This protocol refers to an ongoing study mainly funded by a Brazilian national funding agency (CNPq- 400736/2014-4). The standardized protocol has been implemented in different centers and data collected throughout them. On these occasions, different situations have been experienced and all peculiarities have been recorded in order to help in explaining possible differences among the studied centers.

We expect that the tutored theoretical-practical training will improve the undergraduate students’ performance in the detection of caries lesions and subsequent treatment decisions, mainly in terms of long-term retention of knowledge. Our hypothesis is that tutored theoretical-practical training is a more cost-effective option for teaching undergraduate students to detect caries lesions.

## Discussion

### Motivation and Design

In aiming to formalize the curriculum in Cariology at the 1st Consensus Workshop on the Development of the European Curriculum in Cariology [1], 5 distinct areas were highlighted [2], including the caries diagnosis, which involves risk assessment and detection of caries lesions [16]. However, theoretical classes, often adopted for teaching Cariology, may not be enough to enable those undergraduate students to perform procedures, such as the detection of caries lesions in clinical practice. In this regard, we expect this study to provide the best scientific evidence for defining the best teaching/learning strategy for detection of caries.

As in other areas of dentistry, practical training may be essential for the development of these specific skills that are expected of a future professional [4]. A preliminary study conducted by our research group showed that laboratory activity could help even in solving theoretical questions that the students may have related to caries diagnosis [14]. This is a before and after type of study, in which the same students are evaluated at different times of the laboratory activities. Nevertheless, no study to date has compared this new methodology with theoretical classes. Although the practical activities tend to reinforce the theoretical content, the real cost-efficacy of using this methodology is unknown.

One of the motivations of this multicenter study was checking the impact of the same training activity considering different contexts. The inclusion of several university centers with different students profiles may increase the actual external validity of the proposed activity [13]. Therefore, influence of center dimension, differences in curricula, workflow, faculty formation/willingness to participate in the project, available human resources, and infrastructure/facilities can be tested using this study design. Moreover, distinct universities resources could interfere in the educational outcome or demand an extra investment for implementing the activity.

Most studies that assessed the efficacy or acceptance of students on measures associated with the theoretical classes [12,17,18], but the comparison of these measures with the performance achieved only after theoretical classes is rare [19]. Furthermore, they did not estimate the additional cost for implementation of this alternative, pointing out an innovative approach of our protocol. Once again, the multicenter study design that will permit to compare cost implementation in the different tested contexts [20].

### Expectations

Among positive aspects, active methodologies tend to stimulate higher participation of students in the learning-teaching process [21]. Also, we speculate that by the end of the course undergraduate students could benefit more from this approach than those at the beginning of the course. The cumulative knowledge of the faculty associated with practical training may result in a better performance of dental undergraduate students for application of this integrated knowledge while making decisions related to prevention and management of caries [22].

A recent systematic review [8] has pointed out that the visual caries detection method has good overall performance, and the use of indices improves the accuracy of the method. With the expected results, we aim to achieve the inclusion of the teaching/learning of caries detection using the ICDAS into the curriculum of undergraduate courses. In many clinical disciplines, the content taught in laboratories may have been reduced due to a need to gain time in the clinical environment. On the other hand, considering that laboratory training may help the students solve doubts before clinical examinations and optimize chair time, we believe that tutored theoretical-practical training is a cost-effective option for teaching undergraduate students to detect caries lesions. If the results confirm our hypotheses, this study will contribute significantly to the reformulation of Cariology curriculum guidelines for undergraduate students.

## Appendix

Multimedia Appendix 1 Initiatives for Undergraduate Students’ Training in Cariology collaborative (IuSTC) group.

Multimedia Appendix 2 Funding agency report.

Multimedia Appendix 3 Funding agency report 2.

## Abbreviations

ICDAS: International Caries Detection and Assessment System
