# Supplementary material for: Impact of a Tutored Theoretical-Practical Training to Develop Undergraduate Students’ Skills for the Detection of Caries Lesions: Study Protocol for a Multicenter Controlled Randomized Study
Source: JMIR Res Protoc. 2017 Aug 16;6(8):e155. doi: 10.2196/resprot.7414 (PMC5577444; doi:10.2196/resprot.7414)
Supplement: Multimedia Appendix 1 [file resprot_v6i8e155_app1.pdf]

Additional file

Initiatives for Undergraduate Students' Training in Cariology collaborative (IuSTC) group

Coordinating center:

School of Dentistry, University of São Paulo, São Paulo, Brazil

Principal Investigator: Mariana Minatel Braga

Center coordinators: Fausto Medeiros Mendes, Daniela Prócida Raggio, José Carlos Pettorossi Imparato, Marcelo Bönecker

Technical Support Team:

Alessandra Reyes, Isabela Floriano, Juliana Mattos Silveira, Fernanda Rosche Ferreira, Isaac Murisí Pedroza Uribe, Elizabeth Souza Rocha, Tamara Kerber Tedesco, Karla Mayra Pinto e Carvalho Rezende, Juan Sebastian Lara, Luciana Antonio Pion, Ronilza Matos, Tatiane Fernandes Novaes, Thais Gimenez, Isabella Cristina Louzada, Maria Eduarda Franco Viganó, Raissa Andujas Carlos Pereira, Elisa Abreu Diniz, Caroline Rodrigues da Silva, Raquel Stephani Gomes Guttiérrez, Antonio Carlos Lopes da Silva,

Participant centers:

School of Dentistry, University of São Paulo, Bauru, Brazil
Coordinators: Ana Carolina Magalhães, Linda Wang, Daniela Rios

Araçatuba Dental School, UNESP, Araçatuba, Brazil,
Coordinators: Juliano Pelim Pessan, Cristiane Duque

School of Dentistry, Federal University of Amazonas
Coordinators: Maria Augusta Bessa Rebelo, Ary de Oliveira Alves Filho

School of Dentistry, Postgraduate Programme in Dentistry, Federal University of Piauí
Coordinators: Marina de Deus Moura de Lima, Marcoeli Silva de Moura

School of Dentistry "Prof Albino Coimbra Filho", Federal University of Mato Grosso do Sul
Coordinators: Alessandro Diogo De Carli, Mariane Emi Sanabe

Graduate Program in Dentistry, Federal University of Pelotas
Coordinators: Maximiliano Sergio Cenci, Elenara Ferreira de Oliveira

Dental School, Federal University of Santa Maria
Coordinators: Tathiane Lenzi, Rachel de Oliveira Rocha, Júlio Eduardo do Amaral Zenkner

Universidad de Guadalajara
Coordinator: Isaac Murisí Pedroza Uribe

Universidad El Bosque – Escuela Colombiana de Medicina
Coordinator: Stefania Martignon, Juan Sebastian Lara

Facultad Autonoma de Asunción
Coordinator: Alfredo Carrillo

Universidad Catolica "Nuestra Señora de la Asunción" (Paraguay)
Fatima Gabriela Aquino

Faculty of Dentistry, University of Hong Kong
Coordinators: Chung Hung Chu

University of Sheffield
Coordinator: Chris Deery

University of Dundee
Coordinator: David Ricketts

Faculdade de Medicina Dentária, Universidade do Porto
Coordinator: Paulo Melo

Department of Odontology, University of Copenhagen

Coordinator:Kim Rud Ekstrand
