# Supplementary material for: Impact of a Tutored Theoretical-Practical Training to Develop Undergraduate Students’ Skills for the Detection of Caries Lesions: Study Protocol for a Multicenter Controlled Randomized Study
Source: JMIR Res Protoc. 2017 Aug 16;6(8):e155. doi: 10.2196/resprot.7414 (PMC5577444; doi:10.2196/resprot.7414)
Supplement: Multimedia Appendix 2 [file resprot_v6i8e155_app2.pdf]

CNPq

Plataforma Integrada
Carlos Chagas

Resultado Final

Identificação da Proposta

Número do Processo: 400736/2014-4 **Solicitante:** Mariana Minatel Braga

Chamada: PVE_2014

Título do Projeto: Impacto do treinamento teórico-laboratorial mediado por tutores no desenvolvimento de habilidades de alunos de graduação para detecção de lesões de cárie: estudo multicêntrico controlado e randomizado

Parecer de Deliberação Final

Nota Final

Nota	Ordem	Prioridade
9,74		

Resultado da Avaliação

Favorável

Justificativa:

Pesquisador visitante é referência internacional.. Proposta relevante (cáries).

Recursos

Capital	Custeio	Bolsa	Valor Total
R$ 0,00	R$ 161.685,02	R$ 132.301,74	R$ 293.986,76

Data de Emissão

Data de Emissão do Parecer: 29/08/2014
