# Supplementary material for: Impact of a Tutored Theoretical-Practical Training to Develop Undergraduate Students’ Skills for the Detection of Caries Lesions: Study Protocol for a Multicenter Controlled Randomized Study
Source: JMIR Res Protoc. 2017 Aug 16;6(8):e155. doi: 10.2196/resprot.7414 (PMC5577444; doi:10.2196/resprot.7414)
Supplement: Multimedia Appendix 3 [file resprot_v6i8e155_app3.pdf]

IDENTIFICAÇÃO - PROJETO

NÚMERO DO PROCESSO	LINHA DE FOMENTO/CHAMADA
400736/2014-4	Bolsas no País - Ciência sem Fronteiras / CHAMADA DE PROJETOS MEC/MCTI/CAPES/CNPQ/FAPS - BOLSA PESQUISADOR VISITANTE ESPECIAL - PVE 2014
COMITÊ ASSESSOR	
28 - PROGRAMA ESPECIAL DE COOPERACAO INTERNACIONAL/PECI	
ÁREA DE CONHECIMENTO	
Odontopediatria	
MODALIDADE CONTRATAÇÃO	CA DE JULGAMENTO
Individual	28 - PROGRAMA ESPECIAL DE COOPERACAO INTERNACIONAL/PECI

DADOS DO SOLICITANTE

PROPONENTE	CPF	TITULAÇÃO MÁXIMA
Mariana Minatel Braga	195.508.438-66	Doutorado
DATA DE NASCIMENTO	SEXO	E-MAIL
30/10/1980	Feminino	mmbraga@usp.br
END RESIDENCIAL	TELEFONE RESIDENCIAL	NACIONALIDADE
Av. Carlos Weber, 950 - Bloco Visage , Ap.142 - São Paulo SP	11 - 29254034	Brasileiro
FAX		
INSTITUIÇÃO PROPONENTE		
USP - Universidade de São Paulo (Rua da Praça do Relógio, 109 Sao Paulo) gr@usp.br		
ÁREA DE ATUAÇÃO DO PROPONENTE		
- Ciências da Saúde/Odontopediatria<br>- Ciências da Saúde/Odontologia Social e Preventiva<br>- Ciências da Saúde/Cariologia<br>- Ciências da Saúde/Diagnóstico de cárie		

INSTITUIÇÕES - PROJETO

FUNÇÃO	NOME
Parceria/Cooperação	FOB-USP - Faculdade de Odontologia de Bauru - USP
Parceria/Cooperação	UFMS - Universidade Federal de Mato Grosso do Sul
Executora	USP - Universidade de São Paulo
Parceria/Cooperação	UNESP/ARAÇATUBA - Faculdade de Odontologia de Araçatuba
Parceria/Cooperação	UFPEL - Universidade Federal de Pelotas
Parceria/Cooperação	UFAM - Universidade Federal do Amazonas
Instituição de Origem	UK - University of Copenhagen
Parceria/Cooperação	UFPI - Universidade Federal do Piauí

DADOS GERAIS - PROJETO

TÍTULO

Impacto do treinamento teórico-laboratorial mediado por tutores no desenvolvimento de habilidades de alunos de graduação para detecção de lesões de cárie: estudo multicêntrico controlado e randomizado

SIGLA	DATA DE INÍCIO	DURAÇÃO
	01/09/2014	36 mês(es)

TEMA:

Ciências da Saúde

PALAVRAS CHAVE	HOME PAGE DO PROJETO
cárie dentária; ensino; detecção de lesões de cárie; tutores.; prática laboratorial; treinamento;	

RESUMO

Este estudo multicêntrico tem por objetivo avaliar o impacto da implementação do treinamento teórico-laboratorial monitorado no ensino e treinamento para detecção de lesões de cárie entre alunos de graduação quando comparado ao método de ensino convencional baseado em aulas teóricas expositivas. O impacto dessa atividade de ensino-aprendizagem será medido pela efetividade, custo-benefício, retenção de conhecimento/competências adquiridos e aceitabilidade da atividade pelos alunos de graduação. Para isso, quatorze centros (8 nacionais e 8 internacionais) estão envolvidos na inclusão de sujeitos de pesquisa. Será conduzido um estudo controlado randomizado de grupos paralelos, no qual um dos grupos receberá apenas a aula teórica convencional, com duração entre 60 e 90 minutos e o outro, receberá a mesma aula teórica e também um treinamento teórico-laboratorial com uma hora e meia de duração, envolvendo exercícios e discussões baseadas na avaliação de imagens pré-selecionadas e em exame de dentes extraídos. Os desfechos acima serão avaliados imediatamente após a atividade didática e também em médio e longo prazo. A título de comparação para os desfechos de longo prazo, serão coletados dados de alunos formados em turmas que não passaram pela experiência de treinamento, caracterizando uma etapa de estudo não randomizada, aninhada no estudo principal. Análises estatísticas apropriadas serão realizadas para responder as perguntas inicialmente propostas pelo estudo. Dentro do contexto desse estudo, incluímos a vinda do Pesquisador Visitante Especial, Prof. Kim Ekstrand. Ele será um dos coordenadores de centro dessa pesquisa e participou do desenvolvimento e experimentação de índices para detecção de lesões de cárie, além de ser parte integrante de um Comitê que vem trabalhando para a universalização de um sistema para essa finalidade. Ele contribuirá para a implementação do estudo nos centros nacionais, já que o mesmo tem experiência não apenas no tema, mas também na didática implementada nesse tipo de atividade, que já é praticada por ele há anos em sua Universidade. Além disso, haverá contribuição para os alunos e programas de pós-graduação de todas as instituições nacionais, que poderão participar de atividades como aulas e seminários realizadas pelo professor para a comunidade científica.

ETAPAS / ATIVIDADES

DESCRIÇÃO	INICIO	PRAZO PREVISTO	CONCLUSÃO
Planejamento inicial entre os centros	1º Mês	2 Meses	2º Mês
Preparo inicial do material para treinamento e avaliação	2º Mês	3 Meses	4º Mês
Treinamento para operacionalização do estudo nos diferentes centros	3º Mês	1 Mês	3º Mês
Inclusão e Alocação dos Sujeitos	5º Mês	17 Meses	21º Mês
Avaliação dos desfechos de interesse imediatos	5º Mês	17 Meses	21º Mês
Avaliação dos desfechos de médio e longo prazo	17º Mês	17 Meses	33º Mês
Coleta de dados de turmas não submetidas ao treinamento (para posterior comparação):	4º Mês	29 Meses	32º Mês
Monitoramento da coleta de dados nos centros (à distância):	5º Mês	31 Meses	35º Mês
Monitoramento da coleta de dados nos centros, reforço do treinamento da equipe e apoio na logística das atividades (presencial):	5º Mês	31 Meses	35º Mês
Análises parciais dos dados: parte 1	10º Mês	3 Meses	12º Mês
Análises parciais dos dados: parte 2	22º Mês	3 Meses	24º Mês
Análises parciais dos dados: parte 3	33º Mês	3 Meses	35º Mês
Análise final dos dados:	35º Mês	2 Meses	36º Mês
Redação dos artigos com [illegible]	10º Mês	4 Meses	13º Mês

resultados parciais: parte 1	10º Mês	4 Meses	13º Mês
Redação dos artigos com resultados parciais: parte 2	22º Mês	4 Meses	25º Mês
Redação relatório final e do artigo de compilação de todos os dados coletados	33º Mês	4 Meses	36º Mês
1a visita professor visitante	6º Mês	2 Meses	7º Mês
2a visita professor visitante	24º Mês	2 Meses	25º Mês
3a visita professor visitante	36º Mês	1 Mês	36º Mês

ÁREAS DO CONHECIMENTO - PROJETO

Odontopediatria

DOCUMENTOS ANEXOS

Carta de Recomendação
Curriculum Vitae
Curriculum Vitae
Curriculum Vitae
Curriculum Vitae
Curriculum Vitae
Curriculum Vitae
Curriculum Vitae
Curriculum Vitae
Curriculum Vitae
Curriculum Vitae
Curriculum Vitae
Curriculum Vitae
Currículo
Projeto de Pesquisa

EQUIPE - PROJETO

NOME	FUNÇÃO NO PROJETO	TITULAÇÃO MÁXIMA	ÁREAS DE ATUAÇÃO	
Isabella Cristina Louzada	Aluno	Ensino Médio (2o grau)	- Ciências da Saúde/Odontologia	
**CPF**	**DATA DE NASCIMENTO**	**PAÍS DE NASCIMENTO**	**EMAIL**	**NACIONALIDADE**
419.198.978-28	30/01/1993	Brasil	isabella.louzada@usp.br	Brasileiro

NOME	FUNÇÃO NO PROJETO	TITULAÇÃO MÁXIMA	ÁREAS DE ATUAÇÃO	
Maria Eduarda Franco Viganó	Aluno	Ensino Médio (2o grau)	- Ciências da Saúde/Odontologia	
**CPF**	**DATA DE NASCIMENTO**	**PAÍS DE NASCIMENTO**	**EMAIL**	**NACIONALIDADE**
410.196.828-41	11/09/1991	Brasil	maria.vigano@usp.br	Brasileiro

NOME	FUNÇÃO NO PROJETO	TITULAÇÃO MÁXIMA	ÁREAS DE ATUAÇÃO
Laura Regina Antunes	Aluno		

Pontes				
CPF	DATA DE NASCIMENTO	PAÍS DE NASCIMENTO	EMAIL	NACIONALIDADE
355.877.368-30	09/04/1989	Brasil	laura.pontes@usp.br	Brasileiro
NOME	FUNÇÃO NO PROJETO	TITULAÇÃO MÁXIMA	ÁREAS DE ATUAÇÃO	
Raissa Andujas Carlos Pereira	Aluno	Ensino Médio (2o grau)		
CPF	DATA DE NASCIMENTO	PAÍS DE NASCIMENTO	EMAIL	NACIONALIDADE
373.222.048-67	05/01/1990	Brasil	raissa.andujas.pereira@usp.br	Brasileiro
NOME	FUNÇÃO NO PROJETO	TITULAÇÃO MÁXIMA	ÁREAS DE ATUAÇÃO	
Ana Carolina Magalhães	Pesquisador Colaborador	Doutorado	- Ciências da Saúde/Odontologia<br>- Ciências da Saúde/Odontopediatria<br>- Ciências da Saúde/Cariologia	
CPF	DATA DE NASCIMENTO	PAÍS DE NASCIMENTO	EMAIL	NACIONALIDADE
218.785.658-50	27/07/1980	Brasil	acm@fob.usp.br	Brasileiro
NOME	FUNÇÃO NO PROJETO	TITULAÇÃO MÁXIMA	ÁREAS DE ATUAÇÃO	
Linda Wang	Pesquisador Colaborador	Doutorado	- Ciências da Saúde/Dentística<br>- Ciências da Saúde/Materiais Odontológicos<br>- Ciências da Saúde/Clínica Integrada	
CPF	DATA DE NASCIMENTO	PAÍS DE NASCIMENTO	EMAIL	NACIONALIDADE
206.414.508-77	03/02/1974	Brasil	wang.linda@uol.com.br	Brasileiro
NOME	FUNÇÃO NO PROJETO	TITULAÇÃO MÁXIMA	ÁREAS DE ATUAÇÃO	
Daniela Rios	Pesquisador Colaborador	Doutorado	- Ciências da Saúde/Odontopediatria<br>- Ciências da Saúde/Odontologia Para Gestantes e Bebês	
CPF	DATA DE NASCIMENTO	PAÍS DE NASCIMENTO	EMAIL	NACIONALIDADE
199.112.108-39	12/01/1975	Brasil	daniriosop@yahoo.com.br	Brasileiro
NOME	FUNÇÃO NO PROJETO	TITULAÇÃO MÁXIMA	ÁREAS DE ATUAÇÃO	
Juliano Pelim Pessan	Pesquisador Colaborador	Doutorado	- Ciências da Saúde/Cariologia<br>- Ciências da Saúde/Mecanismo de Ação do Flúor<br>- Ciências da Saúde/Metabolismo do Flúor	
CPF	DATA DE NASCIMENTO	PAÍS DE NASCIMENTO	EMAIL	NACIONALIDADE
285.464.578-24	16/10/1979	Brasil	jpessan@foa.unesp.br	Brasileiro
NOME	FUNÇÃO NO PROJETO	TITULAÇÃO MÁXIMA	ÁREAS DE ATUAÇÃO	

Isabela Floriano Nunes Martins	Aluno de Doutorado	Mestrado	- Ciências da Saúde/Odontopediatria<br>- Ciências da Saúde/Clínica Odontológica	
CPF	DATA DE NASCIMENTO	PAÍS DE NASCIMENTO	EMAIL	NACIONALIDADE
007.349.073-38	24/12/1986	Brasil	isabelafloriano@usp.br	Brasileiro

NOME	FUNÇÃO NO PROJETO	TITULAÇÃO MÁXIMA	ÁREAS DE ATUAÇÃO	
Juliana Mattos Silveira	Aluno de Doutorado	Especialização	- Ciências da Saúde/Odontopediatria<br>- Ciências da Saúde/Cariologia<br>- Ciências da Saúde/Prevenção	
CPF	DATA DE NASCIMENTO	PAÍS DE NASCIMENTO	EMAIL	NACIONALIDADE
041.080.219-08	10/11/1983	Brasil	jmsilveira@usp.br	Brasileiro

NOME	FUNÇÃO NO PROJETO	TITULAÇÃO MÁXIMA	ÁREAS DE ATUAÇÃO	
Fernanda Rosche Ferreira	Aluno de Mestrado	Curso de curta duração		
CPF	DATA DE NASCIMENTO	PAÍS DE NASCIMENTO	EMAIL	NACIONALIDADE
228.028.548-79	05/04/1988	Brasil	fe_rosche@hotmail.com	Brasileiro

NOME	FUNÇÃO NO PROJETO	TITULAÇÃO MÁXIMA	ÁREAS DE ATUAÇÃO	NACIONALIDADE
Isaac Murisi Pedroza Uribe	Aluno de Mestrado			Estrangeiro
CPF	DATA DE NASCIMENTO	PAÍS DE NASCIMENTO	HOME PAGE CV	EMAIL
	08/03/1983	México	Isaac Murisi Pedroza Uribe	

NOME	FUNÇÃO NO PROJETO	TITULAÇÃO MÁXIMA	ÁREAS DE ATUAÇÃO	
Tamara Kerber Tedesco	Aluno de Doutorado	Mestrado	- Ciências da Saúde/Odontopediatria<br>- Ciências da Saúde/Materiais Odontológicos<br>- Ciências da Saúde/Cariologia	
CPF	DATA DE NASCIMENTO	PAÍS DE NASCIMENTO	EMAIL	NACIONALIDADE
008.416.200-75	02/05/1986	Brasil	tamarakt@usp.br	Brasileiro

NOME	FUNÇÃO NO PROJETO	TITULAÇÃO MÁXIMA	ÁREAS DE ATUAÇÃO	
Karla Mayra Pinto e Carvalho Rezende	Aluno de Doutorado	Especialização	- Ciências da Saúde/Células-tronco<br>- Ciências da Saúde/Histologia e Embriologia<br>- Ciências da Saúde/Odontopediatria	
CPF	DATA DE NASCIMENTO	PAÍS DE NASCIMENTO	EMAIL	NACIONALIDADE
293.702.668-01	22/06/1981	Brasil	karla.rezende@usp.br	Brasileiro

NOME	FUNÇÃO NO PROJETO	TITULAÇÃO MÁXIMA	ÁREAS DE ATUAÇÃO	NACIONALIDADE
Juan Sebastian Lara Romero	Aluno de Doutorado	Mestrado	- Ciências da Saúde/Odontologia<br>- Ciências da Saúde/CARIOLOGY	Estrangeiro
**CPF**	**DATA DE NASCIMENTO**	**PAÍS DE NASCIMENTO**	**HOME PAGE CV**	**EMAIL**
234.927.578-71	04/04/1984	Colômbia	Juan Sebastian Lara Romero	juansebastianlara@yahoo.com

NOME	FUNÇÃO NO PROJETO	TITULAÇÃO MÁXIMA	ÁREAS DE ATUAÇÃO	
Luciana Pion Antonio	Aluno	Especialização - Residência médica		
**CPF**	**DATA DE NASCIMENTO**	**PAÍS DE NASCIMENTO**	**EMAIL**	**NACIONALIDADE**
359.012.808-90	31/12/1987	Brasil	lucianapion@hotmail.com	Brasileiro

NOME	FUNÇÃO NO PROJETO	TITULAÇÃO MÁXIMA	ÁREAS DE ATUAÇÃO	
Elenara Ferreira de Oliveira	Pesquisador Colaborador	Doutorado	- Ciências da Saúde/Cariologia<br>- Ciências da Saúde/Dentística	
**CPF**	**DATA DE NASCIMENTO**	**PAÍS DE NASCIMENTO**	**EMAIL**	**NACIONALIDADE**
368.961.100-82	16/07/1955	Brasil	f.elenara@gmail.com	Brasileiro

NOME	FUNÇÃO NO PROJETO	TITULAÇÃO MÁXIMA	ÁREAS DE ATUAÇÃO	
Maximiliano Sérgio Cenci	Pesquisador Colaborador	Doutorado	- Ciências da Saúde/Clínica Odontológica<br>- Ciências da Saúde/Cariologia<br>- Ciências Biológicas/Microbiologia<br>- Ciências da Saúde/Materiais Odontológicos<br>- Ciências da Saúde/Odontopediatria	
**CPF**	**DATA DE NASCIMENTO**	**PAÍS DE NASCIMENTO**	**EMAIL**	**NACIONALIDADE**
699.901.230-04	04/01/1978	Brasil	cencims@gmail.com	Brasileiro

NOME	FUNÇÃO NO PROJETO	TITULAÇÃO MÁXIMA	ÁREAS DE ATUAÇÃO	NACIONALIDADE
Chung Hung Chu	Pesquisador Colaborador			Estrangeiro
**CPF**	**DATA DE NASCIMENTO**	**PAÍS DE NASCIMENTO**	**HOME PAGE CV**	**EMAIL**
	08/05/1964	China	Chung Hung Chu	

NOME	FUNÇÃO NO PROJETO	TITULAÇÃO MÁXIMA	ÁREAS DE ATUAÇÃO	
Quéren Ferreira da Rosa	Aluno	Graduação		
**CPF**	**DATA DE NASCIMENTO**	**PAÍS DE NASCIMENTO**	**EMAIL**	**NACIONALIDADE**

073.607.436-80	21/10/1986	Brasil	querenferreira@yahoo.com.br	Brasileiro

NOME	FUNÇÃO NO PROJETO	TITULAÇÃO MÁXIMA	ÁREAS DE ATUAÇÃO	NACIONALIDADE
David Ricketts	Pesquisador Colaborador			Estrangeiro
**CPF**	**DATA DE NASCIMENTO**	**PAÍS DE NASCIMENTO**	**HOME PAGE CV**	**EMAIL**
	01/03/1958	Gales	David Ricketts	

NOME	FUNÇÃO NO PROJETO	TITULAÇÃO MÁXIMA	ÁREAS DE ATUAÇÃO	NACIONALIDADE
Leticia Ito	Aluno			Estrangeiro
**CPF**	**DATA DE NASCIMENTO**	**PAÍS DE NASCIMENTO**	**HOME PAGE CV**	**EMAIL**
	30/07/1963	China	Leticia Ito	

NOME	FUNÇÃO NO PROJETO	TITULAÇÃO MÁXIMA	ÁREAS DE ATUAÇÃO	NACIONALIDADE
Sofia Jacome Lievano	Pesquisador Colaborador			Estrangeiro
**CPF**	**DATA DE NASCIMENTO**	**PAÍS DE NASCIMENTO**	**HOME PAGE CV**	**EMAIL**
	06/09/1959	Colômbia	Sofia Jacome Lievano	

NOME	FUNÇÃO NO PROJETO	TITULAÇÃO MÁXIMA	ÁREAS DE ATUAÇÃO	NACIONALIDADE
Stefania Martignon	Pesquisador Colaborador			Estrangeiro
**CPF**	**DATA DE NASCIMENTO**	**PAÍS DE NASCIMENTO**	**HOME PAGE CV**	**EMAIL**
	19/05/1967	Colômbia	Stefania Martignon	

NOME	FUNÇÃO NO PROJETO	TITULAÇÃO MÁXIMA	ÁREAS DE ATUAÇÃO	
Kevin Bruce Hall	Aluno	Graduação	- Ciências da Saúde/Odontopediatria<br>- Ciências da Saúde/Cariologia<br>- Ciências da Saúde/Prevenção	
**CPF**	**DATA DE NASCIMENTO**	**PAÍS DE NASCIMENTO**	**EMAIL**	**NACIONALIDADE**
320.222.298-86	24/06/1990	Brasil	kevin_bruce_@hotmail.com	Brasileiro

NOME	FUNÇÃO NO PROJETO	TITULAÇÃO MÁXIMA	ÁREAS DE ATUAÇÃO	
Cristiane Duque	Pesquisador Colaborador	Doutorado	- Ciências da Saúde/Odontopediatria<br>- Ciências da Saúde/Microbiologia<br>- Ciências da Saúde/Endodontia<br>- Ciências da Saúde/Periodontia<br>- Ciências Biológicas/Imunologia<br>- Ciências Biológicas/Genética	
**CPF**	**DATA DE NASCIMENTO**	**PAÍS DE NASCIMENTO**	**EMAIL**	**NACIONALIDADE**
250.468.218-26	04/11/1977	Brasil	cristianeduque@yahoo.com.br	Brasileiro

NOME	FUNÇÃO NO PROJETO	TITULAÇÃO MÁXIMA	ÁREAS DE ATUAÇÃO	
Maria Augusta Bessa Rebelo	Pesquisador Colaborador	Doutorado	- Ciências da Saúde/Cariologia<br>- Ciências da Saúde/Diagnóstico Bucal	
CPF	DATA DE NASCIMENTO	PAÍS DE NASCIMENTO	EMAIL	NACIONALIDADE
136.374.012-15	24/07/1959	Brasil	augusta@ufam.edu.br	Brasileiro

NOME	FUNÇÃO NO PROJETO	TITULAÇÃO MÁXIMA	ÁREAS DE ATUAÇÃO	
Mariana Emi Nagata	Aluno	Especialização - Residência médica	- Ciências da Saúde/Odontologia	
CPF	DATA DE NASCIMENTO	PAÍS DE NASCIMENTO	EMAIL	NACIONALIDADE
044.132.189-57	27/03/1988	Brasil	marieminagata@gmail.com	Brasileiro

NOME	FUNÇÃO NO PROJETO	TITULAÇÃO MÁXIMA	ÁREAS DE ATUAÇÃO	
Marina de Deus Moura de Lima	Pesquisador Colaborador	Doutorado	- Ciências da Saúde/Odontologia<br>- Ciências da Saúde/Ortodontia<br>- Ciências da Saúde/Patologia Bucal	
CPF	DATA DE NASCIMENTO	PAÍS DE NASCIMENTO	EMAIL	NACIONALIDADE
855.656.523-91	30/05/1980	Brasil	mdmlima@gmail.com	Brasileiro

NOME	FUNÇÃO NO PROJETO	TITULAÇÃO MÁXIMA	ÁREAS DE ATUAÇÃO	
Ary de Oliveira Alves Filho	Pesquisador Colaborador	Mestrado	- Ciências da Saúde/Odontopediatria<br>- Ciências da Saúde/Odontologia Social e Preventiva	
CPF	DATA DE NASCIMENTO	PAÍS DE NASCIMENTO	EMAIL	NACIONALIDADE
749.620.192-87	22/03/1983	Brasil	draryfilho@globo.com	Brasileiro

NOME	FUNÇÃO NO PROJETO	TITULAÇÃO MÁXIMA	ÁREAS DE ATUAÇÃO	
Alessandro Diogo de Carli	Pesquisador Colaborador	Doutorado		
CPF	DATA DE NASCIMENTO	PAÍS DE NASCIMENTO	EMAIL	NACIONALIDADE
902.642.070-68	03/01/1976	Brasil	alessandrodecarli@hotmail.com	Brasileiro

NOME	FUNÇÃO NO PROJETO	TITULAÇÃO MÁXIMA	ÁREAS DE ATUAÇÃO	
Marcoeli Silva de Moura	Pesquisador Colaborador	Doutorado	- Ciências da Saúde/Odontologia<br>- Ciências da Saúde/Odontopediatria<br>- Ciências da Saúde/Prevenção<br>- Ciências da Saúde/Odontologia Social e Preventiva	
CPF	DATA DE NASCIMENTO	PAÍS DE NASCIMENTO	EMAIL	NACIONALIDADE

411.691.583-15	30/10/1967	Brasil	marcoeli-moura@uol.com.br	Brasileiro
**NOME**	**FUNÇÃO NO PROJETO**	**TITULAÇÃO MÁXIMA**	**ÁREAS DE ATUAÇÃO**	**NACIONALIDADE**
Lina Maria Marin	Pesquisador Colaborador			Estrangeiro
**CPF**	**DATA DE NASCIMENTO**	**PAÍS DE NASCIMENTO**	**HOME PAGE CV**	**EMAIL**
	18/01/1985	Colômbia	Lina Maria Marin	
**NOME**	**FUNÇÃO NO PROJETO**	**TITULAÇÃO MÁXIMA**	**ÁREAS DE ATUAÇÃO**	
Tathiane Larissa Lenzi	Pesquisador Colaborador	Doutorado	- Ciências da Saúde/Odontologia<br>- Ciências da Saúde/Odontopediatria<br>- Ciências da Saúde/Materiais Odontológicos<br>- Ciências da Saúde/Cariologia<br>- Ciências da Saúde/Odontologia em Saúde Coletiva	
**CPF**	**DATA DE NASCIMENTO**	**PAÍS DE NASCIMENTO**	**EMAIL**	**NACIONALIDADE**
007.039.200-57	04/01/1984	Brasil	tathilenzi@usp.br	Brasileiro
**NOME**	**FUNÇÃO NO PROJETO**	**TITULAÇÃO MÁXIMA**	**ÁREAS DE ATUAÇÃO**	**NACIONALIDADE**
Alfredo Canela Carrillo	Aluno de Mestrado	Graduação	- Ciências da Saúde/Odontologia	Estrangeiro
**CPF**	**DATA DE NASCIMENTO**	**PAÍS DE NASCIMENTO**	**HOME PAGE CV**	**EMAIL**
	01/09/1976	Paraguai	Alfredo Canela Carrillo	odontopediatra2001@hotmail.com
**NOME**	**FUNÇÃO NO PROJETO**	**TITULAÇÃO MÁXIMA**	**ÁREAS DE ATUAÇÃO**	**NACIONALIDADE**
Fátima Gabriela Aquino Barreto	Aluno de Mestrado	Graduação	- Ciências da Saúde/Odontologia	Estrangeiro
**CPF**	**DATA DE NASCIMENTO**	**PAÍS DE NASCIMENTO**	**HOME PAGE CV**	**EMAIL**
233.599.828-51	13/05/1977	Paraguai	Fátima Gabriela Aquino Barreto	faquinobarreto@hotmail.com
**NOME**	**FUNÇÃO NO PROJETO**	**TITULAÇÃO MÁXIMA**	**ÁREAS DE ATUAÇÃO**	**NACIONALIDADE**
Paulo Rui Galrão Ribeiro de Melo	Pesquisador Colaborador			Estrangeiro
**CPF**	**DATA DE NASCIMENTO**	**PAÍS DE NASCIMENTO**	**HOME PAGE CV**	**EMAIL**
	19/07/1962	Portugal	Paulo Rui Galrão Ribeiro de Melo	
**NOME**	**FUNÇÃO NO PROJETO**	**TITULAÇÃO MÁXIMA**	**ÁREAS DE ATUAÇÃO**	**NACIONALIDADE**
Carina Raquel Lemos Coelho	Aluno			Estrangeiro

CPF	DATA DE NASCIMENTO	PAÍS DE NASCIMENTO	HOME PAGE CV	EMAIL
	27/03/1982	Portugal	Carina Raquel Lemos Coelho	

NOME	FUNÇÃO NO PROJETO	TITULAÇÃO MÁXIMA	ÁREAS DE ATUAÇÃO
Fausto Medeiros Mendes	Pesquisador Colaborador	Doutorado	- Ciências da Saúde/Odontopediatria<br>- Ciências da Saúde/Cariologia

CPF	DATA DE NASCIMENTO	PAÍS DE NASCIMENTO	EMAIL	NACIONALIDADE
163.555.778-07	26/05/1974	Brasil	fmmendes@usp.br	Brasileiro

NOME	FUNÇÃO NO PROJETO	TITULAÇÃO MÁXIMA	ÁREAS DE ATUAÇÃO	NACIONALIDADE
Kim Rud Ekstrand	Pesquisador Visitante Especial			Estrangeiro

CPF	DATA DE NASCIMENTO	PAÍS DE NASCIMENTO	HOME PAGE CV	EMAIL
	25/02/1957	Dinamarca	Kim Rud Ekstrand	

NOME	FUNÇÃO NO PROJETO	TITULAÇÃO MÁXIMA	ÁREAS DE ATUAÇÃO
Mariana Minatel Braga	Coordenador	Doutorado	- Ciências da Saúde/Odontopediatria<br>- Ciências da Saúde/Odontologia Social e Preventiva<br>- Ciências da Saúde/Cariologia<br>- Ciências da Saúde/Diagnóstico de cárie

CPF	DATA DE NASCIMENTO	PAÍS DE NASCIMENTO	EMAIL	NACIONALIDADE
195.508.438-66	30/10/1980	Brasil	mmbraga@usp.br	Brasileiro

NOME	FUNÇÃO NO PROJETO	TITULAÇÃO MÁXIMA	ÁREAS DE ATUAÇÃO
Alessandra Reyes	Aluno de Doutorado	Mestrado	- Ciências da Saúde/HOMEOPATIA<br>- Ciências da Saúde/Clínica Odontológica<br>- Ciências da Saúde/Odontopediatria<br>- Ciências da Saúde/HOMEOPATIA<br>- Ciências da Saúde/Clínica Odontológica<br>- Ciências da Saúde/Odontopediatria

CPF	DATA DE NASCIMENTO	PAÍS DE NASCIMENTO	EMAIL	NACIONALIDADE
193.774.008-05	30/09/1974	Brasil	alereyes@ig.com.br	Brasileiro

NOME	FUNÇÃO NO PROJETO	TITULAÇÃO MÁXIMA	ÁREAS DE ATUAÇÃO
José Carlos Pettorossi Imparato	Pesquisador Colaborador	Doutorado	- Ciências da Saúde/Odontopediatria<br>- Ciências da Saúde/Radiologia

CPF	DATA DE NASCIMENTO	PAÍS DE NASCIMENTO	EMAIL	NACIONALIDADE
014.515.428-95	14/12/1964	Brasil	jimparato@usp.br	Brasileiro

NOME	FUNÇÃO NO	TITULAÇÃO	ÁREAS DE ATUAÇÃO

NOME	FUNÇÃO NO PROJETO	TITULAÇÃO MÁXIMA	ÁREAS DE ATUAÇÃO	
Marcelo José Strazzeri Bönecker	Pesquisador Colaborador	Doutorado	- Ciências da Saúde/Odontopediatria<br>- Ciências da Saúde/Epidemiologia<br>- Ciências da Saúde/teleodontologia	
CPF	DATA DE NASCIMENTO	PAÍS DE NASCIMENTO	EMAIL	NACIONALIDADE
093.876.128-56	17/07/1964	Brasil	bonecker@usp.br	Brasileiro

NOME	FUNÇÃO NO PROJETO	TITULAÇÃO MÁXIMA	ÁREAS DE ATUAÇÃO	
Daniela Prócida Raggio	Pesquisador Colaborador	Doutorado	- Ciências da Saúde/Odontopediatria<br>- Ciências da Saúde/Materiais Odontológicos<br>- Ciências da Saúde/Odontologia Social e Preventiva	
CPF	DATA DE NASCIMENTO	PAÍS DE NASCIMENTO	EMAIL	NACIONALIDADE
165.996.058-42	13/10/1972	Brasil	danielar@usp.br	Brasileiro

CUSTEIO CAPITAL

SOLICITADO AO CNPQ

ITEM DE DISPÊNDIO	DESCRIÇÃO	VALOR TOTAL
Custeio	1) Materiais a serem utilizados na implementação das atividades nos centros, estimando alocação de uma média de 130 alunos dos primeiros e últimos anos por centro por ano de inclusão - materiais necessários para preparo das imagens e amostras de dentes a serem usados nos treinamentos e na avaliação do desempenho dos alunos. 2) serviços de terceiros (para revisão de idioma inglês dos artigos a serem publicados e para hospedagem do questionário a ser aplicado para alunos formados)	R$44,054.00
Passagem projeto	Duas idas do coordenador ou um representante a cada um dos centros com média de 4 dias de estadia no local para treinamento e calibração da equipe do centro parceiro para implementar a atividade e para monitoramento/análise preliminar dos dados coletados.	R$42,493.02
Auxílio Deslocamento PVE - 3º ano	Passagem aérea Copenhagen - SP- Copenhagen	R$4,590.00
Auxílio Deslocamento PVE - 1º ano	Passagem aérea Copenhagen - SP- Copenhagen	R$4,590.00
Auxílio Deslocamento PVE - 2º ano	Passagem aérea Copenhagen - SP- Copenhagen	R$4,590.00
Diárias	Duas idas do coordenador ou um representante a cada um dos centros com média de 4 dias de estadia no local para treinamento e calibração da equipe do centro parceiro para implementar a atividade e para monitoramento/análise preliminar dos dados coletados + Vinda de um membro da equipe de cada centro para o centro coordenador para organização preliminar do projeto multicêntrico e treinamento inicial para realização das etapas do projeto.	R$61,368.00

RECURSOS BOLSAS

SOLICITADO AO CNPQ

ITEM DE DISPÊNDIO	DATA DA IMPLANTAÇÃO	QUANTIDADE DE RECURSOS	VALOR TOTAL
Pesquisador Visitante Especial - PVE	01/09/2014	1	R$56,000.00

BENEFÍCIOS

TIPO	QUANTIDADE	VALOR UNITÁRIO	VALOR TOTAL

Mensalidade	4	R$14,000.00	R$56,000.00

ITEM DE DISPÊNDIO	DATA DA IMPLANTAÇÃO	QUANTIDADE DE RECURSOS	VALOR TOTAL
Doutorado Sandwich no Exterior - SWE	01/09/2014	1	R$28,956.19

BENEFÍCIOS

TIPO	QUANTIDADE	VALOR UNITÁRIO	VALOR TOTAL
Auxílio Instalação	1	R$2,929.42	R$2,929.42
Taxa Escolar	1	R$2,253.40	R$2,253.40
Seguro Saúde	4	R$202.81	R$811.22
Mensalidade	4	R$2,929.42	R$11,717.68
Taxa de Bancada	1	R$901.36	R$901.36
Auxílio Deslocamento	1	R$10,343.11	R$10,343.10

ITEM DE DISPÊNDIO	DATA DA IMPLANTAÇÃO	QUANTIDADE DE RECURSOS	VALOR TOTAL
Pós-Doutorado Junior - PDJ	01/09/2014	1	R$58,590.00

BENEFÍCIOS

TIPO	QUANTIDADE	VALOR UNITÁRIO	VALOR TOTAL
Taxa de Bancada	12	R$400.00	R$4,800.00
Mensalidade	12	R$4,100.00	R$49,200.00
Passagem	1	R$4,590.00	R$4,590.00

QUADRO GERAL DE ORÇAMENTO

SOLICITADO AO CNPq

ITEM DE DISPÊNDIO	US$	R$	*TOTAL (R$)
Custeio	0.00	44,054.00	44,054.00
Passagem projeto	0.00	42,493.02	42,493.02
Auxílio Deslocamento PVE - 3º ano	0.00	4,590.00	4,590.00
Auxílio Deslocamento PVE - 1º ano	0.00	4,590.00	4,590.00
Auxílio Deslocamento PVE - 2º ano	0.00	4,590.00	4,590.00
Diárias	0.00	61,368.00	61,368.00
Total custeio	0.00	161,685.02	161,685.02
Pesquisador Visitante Especial - PVE	0.00	56,000.00	56,000.00
Doutorado Sandwich no Exterior - SWE	12,850.00	0.00	28,956.19
Pós-Doutorado Junior - PDJ	0.00	58,590.00	58,590.00
Total bolsa	12,850.00	114,590.00	143,546.19
Total Solicitado ao CNPq (Capital + Custeio + Bolsa)	12,850.00	276,275.00	305,231.19

TOTAL GERAL

ITEM DE DISPÊNDIO	US$	R$	*TOTAL (R$)
Total	12,850.00	276,275.00	305,231.19

VALOR DO DÓLAR DE REFERÊNCIA: R$ 2.2534

DECLARAÇÃO

Ao encaminhar este formulário ao CNPq, o solicitante e o beneficiário declaram formalmente ter conhecimento da chamada e das regras e cláusulas que regem a concessão de bolsas no País e se comprometem a cumpri-las integralmente. Declara, também, que o beneficiário já foi contactado e aceitou o convite de permanecer no Brasil por no mínimo vinte e quatro meses. Declaram, ainda, que têm anuência formal da instituição de execução do projeto e que esta está de acordo com as atividades propostas. O solicitante deverá obter todas as declarações correspondentes e mantê-las à disposição do CNPq e sob sua guarda até a aprovação do relatório técnico final do projeto.

(Declaração de acordo com os artigos 297-299 do Código Penal Brasiliero Código Penal Brasileiro).

NOME	CPF
Mariana Minatel Braga	195.508.438-66

Declaração registrada eletronicamente através da internet junto ao CNPq, mediante uso de senha pessoal do solicitante em 22/06/2014 23:11:52, sob o número de protocolo 9054931543992214
